# Microglial Function and Regulation during Development, Homeostasis and Alzheimer’s Disease

**DOI:** 10.3390/cells10040957

**Published:** 2021-04-20

**Authors:** Brad T. Casali, Erin G. Reed-Geaghan

**Affiliations:** Department of Pharmaceutical Sciences, Northeast Ohio Medical University, Rootstown, OH 44272, USA; bcasali@neomed.edu

**Keywords:** microglia, inflammation, Alzheimer’s disease, neurodegenerative diseases, TREM2, neuroinflammation

## Abstract

Microglia are the resident immune cells of the brain, deriving from yolk sac progenitors that populate the brain parenchyma during development. During development and homeostasis, microglia play critical roles in synaptogenesis and synaptic plasticity, in addition to their primary role as immune sentinels. In aging and neurodegenerative diseases generally, and Alzheimer’s disease (AD) specifically, microglial function is altered in ways that significantly diverge from their homeostatic state, inducing a more detrimental inflammatory environment. In this review, we discuss the receptors, signaling, regulation and gene expression patterns of microglia that mediate their phenotype and function contributing to the inflammatory milieu of the AD brain, as well as strategies that target microglia to ameliorate the onset, progression and symptoms of AD.

## 1. Introduction

Microglia are the resident phagocytes of the central nervous system (CNS). In addition to their immunological role in maintaining CNS homeostasis, microglia play vital roles during development and during neurodegenerative diseases. Previously believed to arise from peripheral sources, microglia are now recognized as ontologically distinct macrophage-like cells. Moreover, the discovery of the unique pedigree of microglia has enabled researchers to elucidate and refine the specialized roles microglia play throughout the CNS, not only in development but also in disease. In this review, we will detail the roles microglia play during development, homeostasis and neurodegeneration, along with analysis of the specialized receptors and gene expression signatures which confer specialized microglial functions. Finally, focusing specifically on mouse models of AD, we will summarize the roles of microglia and the potential therapeutics or techniques which target them during this devastating disease.

## 2. Ontogeny and Development of Microglia

Tissue macrophage hematopoiesis arises from common myeloid precursors and occurs in two distinct waves during embryogenesis in rodents: primitive and definitive. At embryonic day 8.5 (E8.5), primitive hematopoiesis in the fetal yolk sac provides erythromyeloid precursors (EMPs) that seed the developing CNS [1]. During the second wave of definitive hematopoiesis occurring by E10.5, the fetal liver provides the bulk of tissue macrophage precursors [1]. Microglia arise exclusively from EMPs that were trafficked from the yolk sac during primitive hematopoiesis and populated the brain [1]. Zebrafish, in contrast, have multiple microglial sources. Specifically, microglia in zebrafish arise from a region analogous to the yolk sac during embryogenesis, and from the ventral wall of the dorsal aorta in adults [2].

While microglia, other tissue macrophages and peripheral monocytes all arise from EMPs [3,4], subtle differences discriminate microglial development from that of peripheral monocytes and other hematopoietic cells. Microglial development is dependent on the transcription factors PU.1 and IRF8 [5,6], as well as SALL1 [7], which initiate gene expression in a stepwise fashion during development [8]. This is in contrast to peripheral monocytes and macrophages which rely upon Myb1 for development [5]. Other receptors or signaling molecules also dictate microglial development and can partially impact survival, such as the cytokines interleukin 34 (IL-34), and colony-stimulating factor-1 (CSF-1) and its receptor CSF-1R [1,7,9]—explained further below. Microglial development and function are also influenced significantly by the microbiome [10]. Microbiome depletion or manipulation through germ-free conditions or antibiotic treatment results in sexually dimorphic effects on pre- and postnatal microglial transcriptional identity and function [11].

Peripheral monocytes contribute little to the microglial population in homeostasis. Adult microglia are defined by a transforming-growth factor-β (TGF-β)-dependent transcriptional signature, discriminating them from peripheral monocytes that invade the brain in certain experimental paradigms [12,13,14]. Microglia are relatively long-lived cells that rarely proliferate, except during certain CNS insults such as during neurodegeneration, where they self-renew and undergo clonal expansion [13,15,16]. Using transgenic mice carrying multicolor reporters, microglia underwent cell division, serving as a clone to replenish the microglial pool during homeostasis and disease, rather than a common microglial stem or progenitor cell serving as the source of new microglia [16].

## 3. Microglial Functions during Development and Homeostasis

The significant role of microglia in synaptogenesis and synaptic plasticity during development is well documented [17,18]. The innate immune system employs complement, a system of proteins and molecules that targets pathogens and other material for immune cell-mediated destruction. In the CNS, microglia produce the bulk of complement-related proteins [17], although astrocytes have also been shown to express complement components [19]. During development, microglia prune synapses through recognition of the complement components C1q or C3, that tag unwanted synapses [17,20]. C1q is converted to C3, and the receptor for the C3 complement protein (C3R; which consists of the myeloid-specific receptor CD11b) is solely expressed by microglia. Mice deficient in C1q, C3 or C3R all demonstrate reduced microglia-dependent engulfment of synapses and show defects in synapse elimination or pruning during development [18,20,21].

Other proteins on microglia restrain precocious synaptic pruning during development or postnatally. These receptors recognize “don’t-eat-me” ligands expressed on neurons [22], such as CD47, which binds microglial receptor signal-regulatory protein α (SIRPα). The CD47-SIRPα signaling axis ultimately blocks the execution of microglial phagocytosis [22,23]. The expression of CD47 and SIRPα is correlated with regions of active pruning during development [23]. Along these lines, mice lacking either receptor show a decreased synaptic number, and CD47 appears to be localized in active synapse regions associated with activity-dependent microglial engulfment of synapses [23].

Microglia also play pivotal roles in maintaining and contributing to the homeostasis of the neurovascular unit of the brain [24]. During development, there is a population of microglia which migrate near and along blood vessels to developing brain regions [25,26]. Microglia closely contact blood vessels in the murine adult brain [27], participate in the formation of new blood vessels in the retina and certain brain regions [28] and maintain contact with the vasculature not covered by astrocytic endfeet into murine adulthood [26].

During CNS development, microglia secrete or produce trophic factors which promote survival or correct the developmental trajectories of many cell types [29]. Insulin-growth factor-1 (IGF-1) promotes survival of certain types of neural progenitor cells as well as oligodendrocyte precursors during embryonic development [30,31], and specific populations of microglia in the white matter express IGF-1 and other genes implicated in lipid regulation associated with the clearance of oligodendrocytes during development of the brain [32,33,34]. Additionally, microglia-specific IGF-1 promotes myelination in oligodendrocytes, where adult mice with microglia devoid of IGF-1 eventually show defects in myelination [33]. Microglia ablation postnatally and into adulthood decreases the oligodendrocytes and oligodendrocyte progenitor cell pool in the murine brain, leading to reduced postnatal myelinogenesis [35]. These findings are suggestive of the importance of microglia and the factors they produce in supporting other glial cell populations of the brain during development and homeostasis.

Other factors directly produced by microglia, including TGF-β and brain-derived neurotrophic factor (BDNF), promote and regulate development of the CNS. TGF-β signaling in microglia promotes both microglial and nervous system development and behaviors in mice [12,36]. TGF-β signaling also upregulates C1q expression during development in the brain, and TGF-β-deficient animals phenocopy complement mutants and possess reduced synaptic engulfment of inputs in the retina [37]. BDNF produced specifically by microglia was found to be critical in promoting the formation of behaviors associated with learning and memory by modulating proteins involved in synaptic plasticity [38]. The absence of microglia during development results in postnatal learning task deficits and synapse formation defects [38,39]. Glutamatergic excitatory synapse function and proteins are diminished if microglia are ablated postnatally [38]. Microglia also regulate other glia cell populations and their functions during development of the CNS. Both oligodendrocyte development and their functional role in remyelination during disease are regulated to some extent by microglia [35,40,41]. The engulfment of synapses during development requires both microglia-specific receptor expression [42,43] and astrocyte-derived IL-33 for neural circuit formation [44].

In addition to microglial-mediated inputs during CNS development, microglia also monitor and modulate neuronal activity in adult rodents. Due in part to microglia-specific receptors, such as the purinergic receptor P2RY12 (see further below), microglia sense levels of metabolites released into the microenvironment by glia and neurons, directly suppressing neuronal activity and firing during homeostasis [17,39]. Mice with manipulations that preclude sensing of metabolites released from neurons possess hyperactive neuronal activity leading to a greater numbers of seizures than their wildtype counterparts [39]. This neuronal feedback through microglial activity not only controls susceptibility to seizures, but also modulates behavior, potentially creating a pivotal regulatory node during neurodegenerative contexts. Microglia also modulate memories through the complement-dependent elimination of synapses, as evidenced by studies demonstrating preserved memory recall in animals with depleted microglia or administered complement inhibitors [45]. Collectively, these findings implicate microglia in the development and regulation of neuronal homeostasis in the CNS.

## 4. Microglia as Immune Sentinels

Despite accounting for only approximately 5% of the cells in the CNS, microglia are the primary immune cell of the CNS. They are responsible for pathogen clearance, neuronal and glial maintenance and immune surveillance. In this section, we will discuss the receptors and immune-specific functions of microglia in the CNS (see Table 1 for an overview of microglia-specific receptors and signaling pathways).

### 4.1. Microglial Receptors Linked to Immune Regulation and Neuronal Control

Microglia express the chemokine receptor CX3CR1, whose ligand CX3CL1 (otherwise known as fractalkine) is expressed by neurons. The CX3CL1-CX3CR1 axis has been proposed to act as a regulatory node to prevent neurotoxicity through the inhibition of microglial activation [86,87]. Microglial activation is associated with a change in structure associated with the release of proinflammatory cytokines—the latter of which perpetuate neurotoxicity, synapse loss or dysregulated neuronal homeostasis [88]. Postnatal mice lacking CX3CR1 show defective microglial recruitment and reduced synaptic engulfment [46], along with diminished survival of Layer V neurons [49]. In adult mice, loss of CX3CR1 reduces synaptic plasticity in the hippocampus and promotes behavioral impairments [48].

Gene expression profiling in rodents revealed that microglia express high levels of the purinergic receptor P2RY12 relative to peripheral immune cells [12,89]. P2RY12 recognizes modified adenosine nucleotides, such as ATP, and enables microglial processes to rapidly respond to changes in the microenvironment, such as during injury or during neuronal homeostasis or feedback [53]. Significantly, the *P2ry12* promoter selectively and robustly targets microglia over other tissue macrophages in lineage-tracing conditional mouse models [90]. The microglia-specific expression of *Entpd1*, the gene encoding the surface enzyme CD39, converts ATP to adenosine to initiate neuronal purinergic signaling, leading to the microglia-dependent suppression of neuronal activity [39]. Interestingly, neurodegenerative diseases are associated with diminished microglial expression of *P2ry12* and *Entpd1*, and mice lacking these genes show enhanced seizures and altered behavior, pointing towards the collective importance of microglia-specific regulation of neuronal activity [39,52]. P2RY12 is also required for specialized ultrastructural regions between microglia and neurons where microglia monitor neuronal activity directly [54]. Mutant P2RY12 animals also show reduced excitotoxicity in the CA1 and altered fear memory [55].

The triggering receptor expressed on myeloid cells-2 (TREM2) protein has increasingly been appreciated as a central player in the regulation of microglial function during development, homeostasis and disease. TREM2 mediates immune-related processes, such as migration, proliferation and survival in myeloid cells, primarily through its association with adaptor and intracellular signaling proteins [91,92]. Ligands for TREM2 appear to be related to modified lipids, lipoproteins and β-amyloid (Aβ) oligomers [92]. Solely expressed on microglia in the CNS [93], TREM2 signaling mediates both the elimination of synapses by microglia and synapse engulfment via astrocytes during development and homeostasis [42,43].

CSF1R is also important for both macrophages and microglia. Microglia are reliant upon CSF1R for survival, and microglia can be efficiently depleted in adult mice with administration of certain CSF1R antagonists [94]. Mice with depleted microglia show otherwise normal cognition and behaviors [94], but these observations change in a neurodegenerative context. Other tissue macrophages also rely to some extent on CSF1R signaling, and mice devoid of CSF1R lack microglia and other tissue macrophages [1]. The absence of IL-34, another ligand for CSF1R, reduces microglial density [9,66,95]. In peripheral macrophages, CSF1R signaling promotes proliferation, survival and migration [63]. TREM2 and CSF1R signaling may coordinate to facilitate microglial survival in certain contexts [58], but it is currently unclear when this may occur.

Microglia also express other pan-macrophage receptors or markers such as CD45, IBA1, CD11b and F4/80 [96]. This fact has made microglia historically difficult to distinguish from peripheral macrophages based on surface-receptor expression alone. However, gene transcription analyses and single-cell sequencing have enabled the discovery of a microglia-specific transcriptional signature (see further below).

### 4.2. Phagocytosis, Inflammation and Microglia Polarization

As is the case with other immune cells such as macrophages, microglia are equipped to rapidly respond to subtle changes in the microenvironment. This is due primarily to the surveillant nature of microglia, which extend highly complex and branched processes expressing sensitive surface receptors that detect extracellular signals on neurons, glia or the brain parenchyma. Upon detection of an activating stimulus, microglia execute various processes closely associated with their role as immune sentinels, such as phagocytosis and the secretion of cytokines or inflammatory mediators [17,96].

Microglia are proficient phagocytes and perform phagocytosis in order to clear pathogens, apoptotic cells, aggregated proteins and lipid-associated debris [17,96,97]. Acting as an “eat-me” signal, exposed phosphatidylserine on the cell membrane of apoptotic cells activates surface receptors on microglia to initiate phagocytosis through rapid cytoskeletal remodeling [22]. Some prominent microglial phagocytic receptors include the MER receptor and AXL tyrosine kinases (MerTK and AXL, respectively), which activate phagocytosis only after binding to their activated cognate receptor on dying cells [98]; TREM2, which has been shown peripherally and centrally to modulate processes associated with inflammation and phagocytosis and synapse elimination [42,43,60]; and CR3, which binds to C3 or C1q, the latter of which decorates surfaces and act as an opsonin [17]. In contrast, microglial phagocytosis of apoptotic cells or debris can be blocked through microglial surface receptor SIRPα which binds to CD47 expressed on neurons, other cells or myelin [23].

Microglia also phagocytose other types of material associated with neurodegenerative diseases and myelin-associated lipids. Phagocytosis triggers the release of proinflammatory cytokines and reactive oxygen species, activating microglia [99,100]. The microglia-specific surface receptor TREM2 also binds material associated with neurodegenerative diseases, leading to downstream phagocytosis [57]. TREM2, MERTK and AXL have all been implicated in myelin debris clearance during certain neurodegenerative diseases [101]. Microglia also appear to be more proficient phagocytes of myelin versus peripheral monocytes [101]. In several mouse models of neurodegenerative diseases, microglial phagocytotic capacity declines with passage of the disease—most likely due to the inflammatory milieu.

Microglia become activated upon stimulation with particular agents. These stimuli induce microglia to take on a specific activation profile associated with the release of cytokines that influence the cellular milieu. Traditionally, these microglial activation profiles were referred to as “M1” and “M2”, comparable to peripheral macrophages. This nomenclature categorized “M1” microglia as those that secrete pro-inflammatory cytokines or mediators, such as IL-1β, TNF-α, IL-6 and reactive oxygen species [102]. In contrast, “M2” microglia secrete cytokines or mediators associated with immune resolution, phagocytosis and wound healing, such as IL-4, IL-13, IL-10 and Arg1 [103]. It is now clear that cytokines classified as anti- or pro-inflammatory do not produce their expected phenotypes in transgenic mouse models of disease. Thus, the general consensus in the field has evolved to discourage the application of the terms “M1 or M2”, since microglia and macrophages can exist as a heterogeneous population of cells with differing states of activation [102,104,105]. It is now known that microglia show a specific gene expression signature differing from these M1- or M2-activation profiles induced in peripheral macrophages [52]. We will therefore not refer to microglia as M1/M2, but reference the specific cytokines involved. Thus, microglia act as specialized and unique immune sentinels of the CNS during both homeostasis and during disease.

## 5. Microglia in Aging and Neurodegenerative Diseases

Microglia regulate pathologies during neurodegenerative diseases such as AD, Parkinson’s disease (PD) and multiple sclerosis (MS) [106,107]. PD is characterized by motor dysfunction and eventual dementia due to the neurodegeneration of dopaminergic neurons in the substantia nigra as a result of the accumulation of misfolded neuronal α-synuclein [108]. MS is a chronic, inflammatory neurological disorder characterized by lesions of demyelinated nerves in the brain and spinal cord—eventually compromising autonomic, sensory, motor and cognitive functions [107]. MS is thought to arise due to defects in myelination or the immune-mediated destruction of myelinated fibers. Where applicable, we will discuss microglia involvement during these diseases, but we will focus most on microglia during AD.

### 5.1. Normal Aging

There is some evidence that aging influences microglial function. Although microglia adopt a specific transcriptional signature during neurodegenerative diseases, aging may impose distinct transcriptomic changes. In general, aging may foster a more pro-inflammatory brain microenvironment, rendering microglia more sensitive to stimuli [100,109]. Aging can also promote a chronic state of inflammation in macrophages in the periphery, otherwise known as “inflammaging”, which may also extend to microglia [109,110,111]. In both humans and rodents, a subset of microglia progressively become laden with lipids during aging [97]. These “lipid-droplet accumulating” microglia show upregulation of pro-inflammatory cytokines, are defective phagocytes and possess a gene signature similar to that driven by innate immune stimuli such as bacterial endotoxins [97]. Some genes upregulated by these microglia are connected to neurodegenerative disorders [97]. In aging humans, microglia downregulate pathways and proteins associated with homeostasis, such as TGF-β [112]. While sharing some overlap, aging microglia show pathways that differ from microglia during neurodegenerative disorders [113]. Generally, aging alters microglia-specific genes associated with activation and phagocytosis throughout most regions of the brain, including areas commonly exhibiting AD pathology [114,115]. Nonetheless, these findings suggest aging imposes microglial phenotypes associated with an overall divergence from homeostatic processes.

### 5.2. Microglia during AD

AD is characterized by cognitive decline due to the accumulation of two pathological hallmarks: extracellular dense-core β-amyloid (Aβ)-containing plaques and intracellular neurofibrillary tangles composed of the hyperphosphorylated microtubule-binding protein tau [116]. These pathological hallmarks create an inflammatory microenvironment characterized by reactive gliosis that propels neurotoxicity and neuron loss [88,116]. According to the Alzheimer’s Association, AD affected more than 5 million Americans in 2020 [117], and limited therapeutics exist to promote disease resolution or diminish symptom severity. Age is the greatest risk factor for AD, though enhanced risk is associated with the expression of certain apolipoprotein (ApoE) isoforms, encoded by the *APOE* gene [118], the immune system, cholesterol homeostasis and microglia. Studies have pointed to the expression of polymorphisms associated with risk genes such as *CLU*, *BIN1*, *ABCA7*, *TREM2* and *CD33* as risk factors for AD [119,120], all tied to microglia and the immune response. Aside from microglia-specific TREM2 and CD33, which collaborate in mouse models of AD to regulate pathology [121], other genes found in genome-wide association studies have been shown to modify pathology in various AD mouse models [121,122,123,124,125]. In human cases of AD, the expression of TREM2 variants was shown to confer enhanced risk for AD [126,127]. The majority of risk genes are implicated in immune and cholesterol regulation, both of which are often perturbed in neurodegenerative diseases [128,129]. Microglia execute pivotal functions and roles which can contribute to disease regulation and progression during AD, discussed below.

### 5.3. Aβ Clearance

One hallmark of AD pathology is the accumulation of extracellular Aβ, which is produced following a series of cleavage events of a precursor protein in neurons by membrane-bound enzymes. Soluble Aβ monomers can accumulate in the extracellular space, eventually fibrillizing into insoluble aggregates and dense-core plaques. Both soluble and insoluble forms of Aβ are cleared through different mechanisms [130,131]. Microglia efficiently promote clearance of both soluble and insoluble forms of Aβ.

Soluble forms of Aβ (sAβ) are chiefly cleared through macropinocytosis in microglia [130]. In this process, sAβ is taken up in microglial cells and targeted for intracellular proteolytic degradation [131]. Degradation of sAβ is enhanced by and dependent upon the lipidation status of ApoE high-density lipoproteins (ApoE-HDLs) [132]. Therapeutics enhancing genes associated with ApoE production and its lipidating receptors produce salutary outcomes in various animal models of neurodegenerative diseases, including AD [131,133]. Microglia have been shown to secrete ApoE-HDLs which differ in their propensity to be lipidated relative to astrocyte-derived ApoE-HDLs [134]. Whether these glial-specific ApoE-HDLs differentially impact neurodegenerative disease is unclear.

The clearance of insoluble or fibrillar Aβ (fAβ) and plaques is mediated solely by microglia via a complex of surface receptors that culminate in the delivery of fAβ to the lysosome for degradation [84,135,136,137,138]. Envelopment of fAβ or plaques induces the release of pro-inflammatory cytokines which may promote neurotoxicity [88]. The presence of anti-inflammatory cytokines or mediators, such as IL-4, augments fAβ phagocytosis, whereas a microenvironment with pro-inflammatory cytokines, such as IL-6, blocks fAβ phagocytosis in microglia [99,139]. Drugs such as nuclear receptor agonists bexarotene, pioglitazone and GW3965 promote the upregulation of scavenger receptors or genes associated with phagocytosis, ameliorating AD pathology in various mouse models [131,133,140,141].

### 5.4. Microglial-Mediated Plaque Barrier Function and Neuroprotection

While microglia become less efficient at phagocytic clearance of Aβ with time, microglia nevertheless maintain contact with plaque borders throughout disease [142]. In humans and in mouse models of AD, microglia form a barrier around dense-core plaques. They remodel plaque perimeters to limit newly formed soluble Aβ from binding to high-affinity Aβ ‘hotspots’—regions containing less Aβ and not covered by microglia processes [143]. Another purpose of the microglia barrier is to impede plaque edges from damaging nearby healthy neurites via direct-contact of the plaque by microglial processes [143]. Overall, the degree of plaque compaction is directly correlated with the degree of plaque-associated neuritic dystrophy [143]. Therefore, microglial barriers appear to be the most efficient at restraining neuritic dystrophy spread around smaller plaques [50]. Inhibition of either CX3CR1, the microglial receptor responsible for chemotaxis and synaptic plasticity, or plaque engagement via Aβ-directed antibodies by microglia, ameliorates plaque compaction and microglial coverage, and reduces neuritic dystrophy [50]. On the other hand, the microglia-specific receptor TREM2, which controls phagocytic processes, is required to form efficient microglial-mediated barriers around plaques in various mouse models of AD [50,61]. Microglial depletion precludes barrier formation but is reversible [144,145]. Thus, microglial-mediated plaque barriers promote neuroprotection in many mouse models of AD.

### 5.5. Inflammation and Polarization

During disease, Aβ is associated with microglial activation, the release of pro-inflammatory cytokines and subsequent neurotoxicity [88,116]. Depending on disease stage, microglial inflammation and polarization may influence disease progression in AD [146]. Broadly, inflammation-associated signaling in microglia can be regulated at the level of surface-receptor expression, or through transcriptional blockade of cytokines and inflammatory mediators. CX3CR1 appears to block microglial activation to mediate neurotoxicity [87], and certain surface receptors, such as TREM2, may be shed from the surface to create a soluble counterpart in order to modify pathology [147]. The mechanisms by which soluble TREM2 regulates inflammation are unclear. Nuclear receptors (explained further below) both inhibit transcription of pro-inflammatory cytokines and promote the upregulation of genes associated with the suppression of inflammation and Aβ clearance [131].

Microglia-specific receptors impact AD pathology and progression in various mouse models. Mice lacking CX3CR1 show reduced plaque burden and neurotoxicity in amyloid-driven mouse models, whereas transgenic mice overexpressing tau show worsened behavior and microtubule-associated pathology [148,149,150,151]. Additionally, CX3CR1 loss promotes neuron loss in mouse models of AD [148]. Microglia progressively lose a gene signature associated with homeostasis upon induction of neurodegenerative diseases, which is marked by downregulation of *Cx3cr1* [51].

TREM2 manipulation, either through genetic deletion or antibody-dependent activation, may have different effects on pathology in various amyloid- and tau-expressing AD mouse models [152,153,154,155,156]. Soluble forms of TREM2 also appear to modulate AD pathology through direct action on microglial phenotypes [157,158]. Nonetheless, TREM2 or TREM2 variants appear to modulate microglial activation. Downstream of TREM2 signaling, the microglia-specific receptor CD33 controls uptake of Aβ in AD mouse models [159], contributing to disease progression [121].

The glycoprotein CD200 is expressed by neurons, glial cells, leukocytes and endothelial cells [73]. Its receptor, CD200R, is expressed chiefly by microglia and myeloid cells. The CD200-CD200R signaling axis delivers inhibitory signals to block microglia activation in the brain and retina [72,73]. Along these lines, CD200 knockout mice show macrophages and microglia with enhanced proinflammatory cytokines and activated phenotypes [74], whereas anti-inflammatory cytokine IL-4 promotes the upregulation of CD200R in microglia [78]. During injury, CD200 is upregulated on neurons, where it may act to inhibit microglia [75,76]. In neurodegenerative diseases, such as AD, CD200 is downregulated in human brains [77], and forced overexpression in the brain of CD200 promotes neurogenesis and ameliorates pathology in a mouse model of AD [79].

Secreted cytokines and proteins derived from microglia can exacerbate disease. Using microglia depletion and knockout mice, studies demonstrated that the microglia-dependent release of cytokines TNFα, IL-6 and the complement protein C1q induce the conversion of quiescent astrocytes into neurotoxic astrocytes displaying a specific gene signature associated with neurodegeneration [71]. Microglia-specific complement proteins and receptors play key roles in exacerbating disease, especially early in disease where C1q promotes Aβ-induced synapse elimination in the hippocampus and subsequent behavioral impairments [70]. C1q promotes oligomeric Aβ-associated neurotoxicity in the hippocampus, and blockade of C3 or C3R signaling retains synapses in an AD mouse model [70]. Although these components facilitate the removal of synapses and neurons by microglia during development and homeostasis, neurodegenerative diseases are associated with the upregulation of C1qa and C3 proteins [17]. This leads to microglial-mediated synapse elimination and neurodegeneration.

Toll-like receptors (TLRs) act as pattern-recognition receptors and detect innate immune stimuli associated with pathogens or cell damage [81]. Activation of these receptors initiates the proliferation, activation and clearance of Aβ in microglia [80]. One prominent example is TLR4, which forms a complex with the Aβ-binding co-receptor CD14 in microglia [82,83]. Microglia lacking TLR4 show hampered phagocytosis of fibrillar Aβ and abrogated release of cytokines [84]. Furthermore, loss-of-function mutations in TLR4 exacerbate Aβ burden and impair microglial activation in mouse models of AD [85], collectively highlighting the importance of TLR activation in AD. The functions and phenotypes associated with other TLRs have been characterized and summarized during homeostasis and disease in the CNS; however, this topic is beyond the scope of this review and detailed elsewhere [80,160,161].

Originally characterized in peripheral macrophages, inflammasomes recognize danger signals associated with the self or toxins, such as oxidized lipoproteins, asbestos, sodium urate crystals associated with gout and fibrillar forms of Aβ [162]. Activation of the inflammasome culminates in cell-mediated pyroptosis, a caspase-dependent type of cell death associated with inflammation [163]. Additionally, subunits of the inflammasome complex can be released from microglia, whereupon they promote enhanced seeding and accumulation of Aβ plaques in an AD mouse model due to their prionoid-like activity [164,165]. Interestingly, both amyloid- and tau-expressing AD mouse models demonstrate ameliorated pathologies when components of the inflammasome are deleted [166,167], pointing to the importance of immune components and mediators in microglia (see Table 1 for a summary of microglia-specific receptors and signaling involvement during AD).

### 5.6. Homeostasis and Neurodegeneration Exhibit Distinct Microglial Gene Signatures

Although microglia and peripheral macrophages execute similar functions, such as cytokine release and phagocytosis of debris and pathogens, microglia possess unique regulatory mechanisms during homeostasis and during disease. Our recent understanding of the transcriptional differences during homeostasis and disease in microglia from that of other tissue phagocytes has been due to the advances of single-cell sequencing and flow cytometric technologies. In this section, we will focus on the transcriptional signatures defining microglia during tissue homeostasis and how this unique transcriptional framework subserves microglia functions during neurodegenerative diseases.

In the non-diseased CNS, microglia exist in a homeostatic state and express a unique transcriptional signature that differs from non-CNS tissue macrophages. This homeostatic signature is defined by high expression levels of surface proteins CX3CR1, P2RY12, TMEM119 and TGF-β receptor 1 (TGFBR1), along with various transcription factors and other genes [96,168]. During neurodegenerative diseases, microglia adopt a collective signature significantly different from the homeostatic transcriptional signature. These “disease-associated microglia” (DAM) acquire a gene signature collectively associated with a “microglial neurodegenerative (MGnD)” phenotype. Although most research has focused on rodent neurodegenerative models, DAM-like cells have been observed in human AD brains [52].

The acquisition of DAM-like cells occurs temporally, with some studies suggesting various stages of DAM based on marker expression alone [51]. The first step coincides with the downregulation of the homeostatic genes *Cx3cr1*, *P2ry12* and *Tmem119*, which encode their respective surface receptors normally expressed at high levels in microglia [51,169]. Some homeostatic receptors provide known inhibitory signaling, as is the case with CX3CR1 and its cognate neuronal ligand CX3CL1, which provides protection against microglial-mediated neurotoxicity [47]. The downregulation of homeostatic genes coincides with the upregulation of specific DAM or MGnD-associated genes, such *Trem2*, *Apoe*, *Lpl*, *Cst7*, *Spp1* and *Clec7a*, among others [96,169]. The majority of these genes are associated with the production of proteins involved in lipid metabolism, phagocytosis, clearance of apoptotic cell bodies and the immune response [96,129,169,170]. Interestingly, the MGnD phenotype is consistent across various neurodegenerative mouse models. DAM-like cells or MGnD phenotypes have been characterized in the Aβ-producing 5xFAD and APP/PSΔE9 AD mouse models [51,171,172], the Tau-producing P301S AD mouse model [173], mouse models of amyotrophic lateral sclerosis [51,52,174] and mouse models for multiple sclerosis [52].

**Figure 1:**
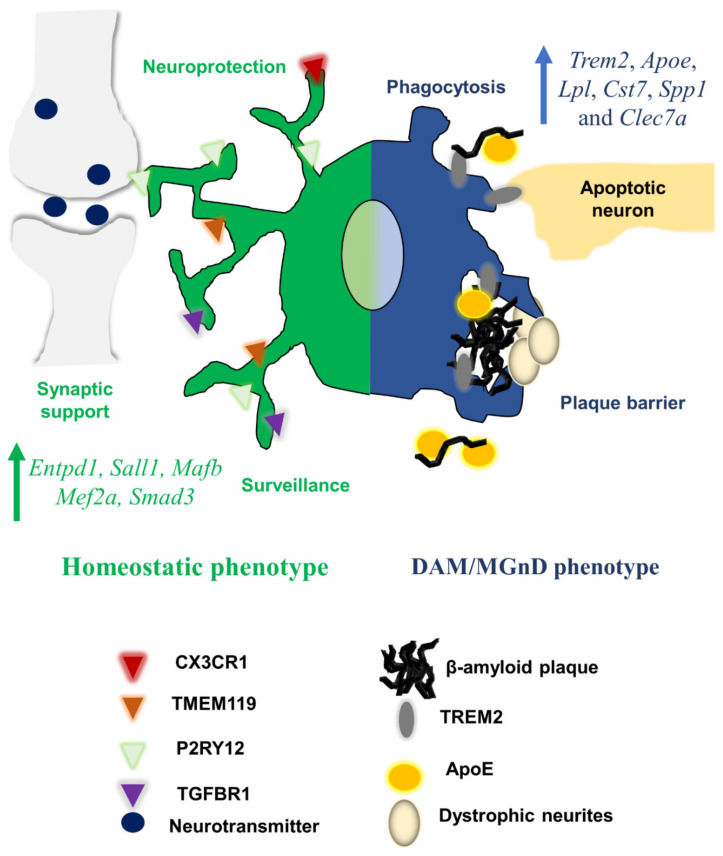
Regulation of the homeostatic and DAM/MGnD phenotypes. The homeostatic phenotype (green) is associated with phenotypes associated with microglial-mediated neuroprotection, synaptic support and immune surveillance. Surface receptors are upregulated in the homeostatic state, such as CX3CR1, TMEM119, P2RY12, TGFBR1 and CD39, encoded by *Entpd1*, all of which facilitate homeostatic responses. Homeostatic microglia display key transcription factors, such as Sall1, Mef2c and Smad3. Onset of neurodegenerative-associated pathology such as Aβ, or apoptotic neurons, triggers downregulation of homeostatic surface receptors and upregulation of the DAM/MGnD phenotype (blue)—the latter associated with markers *Trem2*, *Apoe*, *Lpl*, *Cst7*, *Spp1* and *Clec7a*. Phagocytosis of apoptotic neurons and phagocytosis of Aβ is performed via TREM2- or ApoE-mediated recognition signals in microglia. ApoE and TREM2 coordinate expression of DAM/MGnD genes, as well as facilitate formation of the microglial-mediated plaque barrier to protect neighboring neurons from dystrophic neurite spread.

The expression of certain receptors and transcription factors controls the expression of the homeostatic microglial signature (Figure 1). A key determinant of the microglia-specific gene signature is the expression of *Tgfbr1*, encoding the TGFBR1 that binds TGF-β1. This microglia-specific TGF-β-defined signature is distinct from peripheral tissue macrophages [12], but there are some instances when monocytes may adopt a TGF-β-dependent signature [175]. High expression of *Tgfbr1* is observed in homeostatic microglia [36,52]. Mice devoid of TGF-β1 in the CNS showed reduced numbers of microglia, lacked homeostatic-like microglia signatures and displayed enhanced mortality due to paralysis compared to wildtype littermates [12,36]. Activation of SMAD proteins downstream of TGF-β receptors induces homeostatic-like gene repertoires in microglia [176]. The transcription factor SALL1, whose expression is restricted to microglia compared to other tissue phagocytes, maintains homeostatic microglia, and its deletion leads to impaired neurogenesis and renders microglia more inflammatory and phagocytic [7]. In the adult rodent, certain homeostatic functions are conferred by the expression of transcription factors *Mafb* and *Mef2a*, both of which are induced in a stepwise fashion during development [8]. The transcription factor PU.1, which is highly expressed by myeloid cells and required for microglial development, shows enrichment in binding sites for other microglia homeostatic transcription factors, thereby facilitating microglia-specific signatures [6,177,178,179]. In DAM/MGnD phenotypes, these aforementioned transcription factors are suppressed [96].

The control of microglial homeostasis involves several regulatory nodes in the form of surface proteins and transcription factors. Most of this regulation is due in part to microglial surveillance of the environment. In the neurodegenerative context, some have proposed that microglia sense and respond to neurodegeneration-associated molecular patterns, otherwise known as NAMPs [169]. Molecularly, NAMPs function similarly to pathogen-associated molecular patterns (PAMPs) present on bacteria and viruses. Examples of NAMPs include extracellular protein aggregates composed of Aβ, apoptotic neurons, myelin debris and extracellular lipid degradation products [169]. Certain “classical activation” ligands (e.g., bacterial endotoxins) that polarize peripheral macrophages towards a classic inflammatory gene expression profile do not induce the DAM/MGnD-phenotypic signature in microglia, although minor gene expression overlap does occur [52]. These findings further support the notion that the underlying mechanisms for DAM gene signature acquisition are specific to microglia and could be due to unique microglial ontogeny. On the other hand, DAM cells could be a result of microglial reactivity to misfolded protein aggregates, the latter of which are hallmarks of many neurodegenerative diseases.

DAM cells are often proximal to various NAMPs, whereas microglia further from NAMPs maintain a homeostatic profile. For example, microglia around apoptotic neurons or Aβ-associated dystrophic neurons show high expression of CLEC7A and low levels of the homeostatic protein P2RY12, whereas microglia associated with non-diseased neurons possess the inverse profile [52]. In AD mouse models, the expression of microglia surface receptor CD39 (encoded by *Entpd1*) becomes downregulated, suppressing microglial regulation of neuronal activity [39,52]. Microglia sensing of NAMPs initiates innate immune signaling and concomitant transcriptional activation of the MGnD/DAM phenotype, enabling microglia to block pathological effects from neurodegenerative byproducts through the phagocytosis of Aβ or microglial-mediated barrier formation to promote plaque compaction and lessen plaque-associated neuritic dystrophy, for example [50,61]. Manipulation of CX3CR1 signaling has been shown to abrogate plaque burden in an AD mouse model, though the authors of the original study did not analyze DAM/MGnD phenotypic gene signatures [149]. On the other hand, DAM-like cells may also promote neurodegeneration if microglia lose the ability to maintain homeostatic functions [52]—see Figure 1.

### 5.7. ApoE Regulation of DAM/MGnD Phenotype

Though many genes are altered in the DAM/MGnD phenotype, most advances in our understanding of microglial biology during disease have centered on microglia-specific proteins and ApoE, the greatest risk factor for late-onset AD after age [180]. In particular, the microglia-specific surface protein TREM2 is pivotal in regulating the DAM and MGnD phenotypes. Microglia from TREM2-deficient mice possess a dampened gene activation profile, and fail to migrate to apoptotic or injured neurons [59]. Forced overexpression of TREM2 ameliorates the chemotactic deficiencies [59], pointing towards the critical role TREM2 signaling plays in microglial activation. Interestingly, expression of *Ccl2*, which encodes the chemokine responsible for mediating monocyte and microglia migration through binding to chemokine receptor CCR2, is upregulated in DAM microglia [52]. TREM2-null AD mice lack a DAM signature and MGnD phenotype, and these mice possess enhanced neuroprotection in young mice versus TREM2-expressing AD littermates [51,52]. Additionally, the overexpression of TREM2 abrogates AD pathology via the upregulation of genes involved in phagocytosis, immune regulation and neuroprotection, culminating in reduced plaque burden and diminished neuritic dystrophy in the 5xFAD mouse model [181]. Others have demonstrated that DAM transformation occurs in two steps, with the final stage of DAM acquisition being dependent on TREM2 signaling [51]. Besides binding to Aβ [57], TREM2 also acts to sense levels of various lipoproteins such as ApoE that are found in plaques and could promote plaque seeding [56,58,182,183].

The DAM/MGnD-phenotype is characterized by the robust expression of *Apoe*, which encodes ApoE, and its expression is negatively correlated with that of the homeostatic marker *Tgfb1* in various models of neurodegeneration [52]. Aside from the multifaceted roles ApoE plays in AD [180], its signaling in microglia is currently an active area of research. Microglia that do not express ApoE lack a complete DAM signature, and mice with *Apoe*-deficient microglia also show increased neuroprotection in a model of acute neuronal ablation [52]. Interestingly, MGnD phenotypes were only observed in microglia phagocytosing apoptotic neurons—microglia not phagocytosing apoptotic neurons lacked a complete MGnD phenotype or DAM acquisition [52]. AD mouse models with global *Apoe* deletion possess microglia with reduced activation profiles [52,184]. These mice also lack microglial plaque barriers and exhibit enhanced neuritic dystrophy due to reduced plaque compaction [184]. Along these lines, reduced plaque-associated ApoE and microglial clustering around plaques were observed in AD mice lacking TREM2 [185]. On the other hand, plaque seeding was enhanced in these animals, suggesting that ApoE activates microglia around or near plaques.

Though the underlying signaling mechanisms are still active areas of research, TREM2, in concert with ApoE, plays fundamental roles in controlling DAM and MGnD phenotypes. Nonetheless, it is currently a matter of debate and active research, whether DAM or MGnD phenotypes per se are harmful or beneficial in the context of neurodegenerative diseases [96].

### 5.8. The Role of Myeloid Cells at Brain–Border Interfaces in Neurodegenerative Diseases

The CNS experiences little infiltration from the peripheral immune system during homeostasis; however, the peripheral immune system contributes significantly to certain neurodegenerative diseases, such as in experimental autoimmune encephalomyelitis (EAE) mouse models of MS. These studies have generated considerable controversy about the relative contribution of the peripheral immune system in the CNS during other neurodegenerative diseases. Some work demonstrated that bone marrow-derived monocytes infiltrated the brain during neurodegenerative diseases such as AD [186]; however, these studies utilized either whole-body or brain-shielded irradiation, both of which damage the blood–brain barrier (BBB), promote radiation-dependent gene upregulation in brain-resident microglia [187,188,189,190] and alter the brain’s microenvironment to permit monocyte infiltration [191]. Similarly, other studies have employed genetic models that ablate microglia, triggering an influx of peripheral monocytes into the brain in order study the peripheral immune system during homeostasis and neurodegenerative diseases [13,14,192]. Infiltrating monocytes then differentiated to adopt a ramified, microglia-like morphology upon extravasation into the parenchyma [13,14,192], but these monocytes maintained a transcriptional signature distinct from resident microglia [13]. In contrast, studies using strategies less disruptive to the blood–brain barrier (BBB), including parabiosis or cell-specific lineage tracing, refute that work, instead demonstrating that the infiltration of monocytes into the brain is minimal, even during AD [193,194].

In addition to the microglia that survey the brain parenchyma, myeloid cells exist within the anatomical spaces and regions bordering the brain. The choroid plexus (CP), meninges and dura and pia mater contain myeloid cells collectively referred to as border-associated macrophages (BAMs). The majority of BAM subsets exhibit transcriptional signatures significantly distinct from those of circulating monocytes and parenchymal microglia; however, one known subset of BAM possesses a transcriptional signature similar to that of parenchymal microglia. Collectively, BAMs share a resting gene signature that is distinct from homeostatic microglia, with each subset expressing a unique cadre of genes. All BAMs, however, show high expression levels of *Apoe*, *Ms4a7* and *Lyz2* relative to resting microglia [171]. *Ms4a7* encodes the multipass proteins of the tetraspan MS4A family expressed in macrophages and microglia. MS4A family members have been shown to modulate TREM2 levels and confer enhanced AD risk [195,196]. Epiplexus BAMs, which reside on the CP epithelium, possess a resting gene signature that most closely resembles the DAM/MGnD phenotype, with high expression of lipid and phagocytic-related genes *Apoe*, *Cst7*, *Clec7a* and *Lpl* [171]. Thus, CP epiplexus BAMs most resemble parenchymal microglia. Along these lines, CP epiplexus BAMs and parenchymal microglia can be selectively targeted with the *Sall1* promoter [171,197].

In terms of myeloid-specific transcription factors, nearly all BAMs rely on PU.1 for survival and are independent of other common monocyte-related transcription factors such as Myb1 [198]. Interestingly, when the transcription factor IRF8 is silenced in microglia, this cell population adopts a signature similar to that of BAMs, lending credence to the indispensable role IRF8 plays in microglia-specific ontogeny [171]. IRF8 is required for the development of microglia [6], tissue macrophages [199] and other myeloid cells [200]. Anatomical locations more proximal to the blood, such as the CP and dura, show significant monocyte contribution during steady state; however, nearly all BAMs show some self-renewal capacity [171,198]. Like parenchymal microglia, macrophages at the brain–border interfaces are also susceptible to depletion via small-molecule inhibitors against CSF-1R [94,171,197]. Collectively, these findings suggest that despite unique transcriptional profiles, to some extent, microglia and BAMs rely on common macrophage-specific receptors for survival.

The lymphatics in proximity to the border-associated regions, such as the meninges, are critical gateways for other immune cells to interact with cerebral spinal fluid (CSF) in draining lymph nodes nearest the CNS to influence neurodegenerative diseases. Ablation of meningeal lymphatics ameliorates recovery in a mouse model of MS due to dampened neuroinflammation [201]. In contrast, AD mouse models lacking meningeal lymphatic drainage demonstrate enhanced parenchymal Aβ plaque deposition in the meninges and in the parenchyma [202]. Thus, myeloid cells at anatomical border sites may play different roles compared to parenchymal microglia depending on the type of neurodegenerative disease.

The precise roles BAMs play during aging and neurodegenerative diseases are unresolved. Nonetheless, relative BAM populations shift in aging and mouse models of AD, depending on marker classification [171,197]. In EAE mouse models of MS, T cells and peripheral monocytes represent the bulk of infiltrative immune populations, but BAMs maintain an activating signature distinct from the invading peripheral monocytes [197]. Moreover, macrophages associated with the vasculature self-renew through acute and chronic phases of the disease during EAE progression [198]. Interestingly, CP epiplexus BAMs maintain a DAM/MGnD phenotype in an AD mouse model, and these BAMs exhibit a high phagocytic capacity, probably owing to the fact that the CP stroma directly interfaces with the lipid and lipoprotein-containing CSF [171].

## 6. Targeting Microglia during Development and Disease

Since there are no targeted therapeutics to control or reverse the symptoms of AD, there is immense clinical interest in developing novel drugs to target receptors, pathways or cell groups. While much attention has been given to therapeutics that ameliorate pathology in AD mouse models, the recent advances in the unique microglial biology and function have a greater therapeutic potential for the treatment of AD specifically, and neurodegenerative diseases more broadly. In this section, we will discuss genetic models to target microglia during development and disease as well as specific agents which modulate microglia or microglial functions during disease. Though these models are not yet ready for clinical use and are not likely to be logical therapies themselves, they provide critical avenues by which researchers can study the functions of microglia during development and homeostasis, thereby identifying conducive therapeutic targets and strategies.

### 6.1. Genetic Models to Target Microglia

Since macrophages are phenotypically indistinguishable from microglia, it has been traditionally difficult to selectively target microglia in rodent models. The *LysM*-Cre models, which drive Cre-recombinase under the *Lyz2* promoter, can be used to target subsets of microglia during development. However, the ubiquitous expression of *Lyz2* by peripheral myeloid cells and low targeting efficiency of microglia, as well as expression in neurons, potentially confound its use for microglia-specific targeting [203,204,205]. The chemokine receptor CX3CR1 has been reported to label various tissue macrophages, dendritic cells, monocytes and microglia in knock-in and conditional models crossed to reporter lines [206]. Animals expressing a mutant Cre enzyme fused to a modified estrogen receptor (CreER) can be induced to express fluorescent reporters after tamoxifen administration [206]. With this inducible model, the above populations are targeted, but dendritic cells and circulating monocytes undergo rapid replacement by tamoxifen-naïve precursors relative to microglia, the latter of which do not show appreciable turnover. According to some studies, the fidelity for targeting microglia with the *Cx3cr1*-CreER model approaches or exceeds 90% [206,207]. This model has been successfully used to label and track microglia during development and disease, but also has been utilized to delete genes flanked by loxP sites (“floxed” alleles) solely in microglia. TMEM119, a robust marker for homeostatic microglia, can be utilized to modify or label microglia in transgenic mice, but some fibroblasts and myeloid populations associated with the brain border may also be targeted [208,209]. The homeostatic receptor P2RY12 can also be used to efficiently target microglia [90]. Even still, *Sall1*-CreER targets microglia and a subset of epiplexus CP BAMs [7,171], whereas *Cx3cr1*-CreER and *P2ry12*-CreER target both microglia and nearly all types of BAM cells to some extent [90,198,210]. It is important to note that the expression of these homeostatic markers diminishes during disease progression, and particular attention should be exercised as to the timing of gene deletion or manipulation. Thus, there are various mouse models at the disposal of researchers to target microglia during development and disease, but off-target cell effects should also be considered associated with these models.

### 6.2. Microglia Depletion and Targeting Microglial Receptors during Homeostasis and Disease

Microglia rely on CSF1R signaling in some capacity for survival, as using certain CSF1R inhibitors eliminates microglia in mouse models [28,94]. Depending on the paradigm used, microglial depletion efficiency approaches 98% [94], similar to other genetic strategies to eliminate microglia [211,212]. Nevertheless, microglial depletion may be less efficient in certain disease contexts such as AD [213]. It is unclear whether microglia resistant to depletion rely on other survival signals aside from CSF1R, but evidence strongly suggests other receptors, such as TREM2, may provide compensatory survival pathways [58]. A recent study suggested depletion-resistant microglia may derive from a microglia-like progenitor cell similar to the EMPs from which microglia arise [214]. Additionally, plaque-associated microglia downregulate *Csf1r* upon transition to DAM-like cells [52], and microglia from mice with enhanced plaque burden are resistant to CSF1R-mediated depletion [144,145]. In almost all cases, microglia repopulate through self-renewal following drug removal, exhibiting nearly identical gene signatures and functions relative to their non-depleted counterparts in a homeostatic environment [94,215]. Under certain experimental conditions, peripheral monocytes do contribute to the repopulating microglia pool, but both cell types maintain distinct transcriptional signatures [13,14,216,217]. Microglia depletion during adulthood in mice and rats does not negatively affect behavior or cognition [94,215]. On the other hand, the elimination of microglia embryonically or postnatally alters juvenile and adult anxiety-like behaviors in mice and rats [218,219].

**Figure 2:**
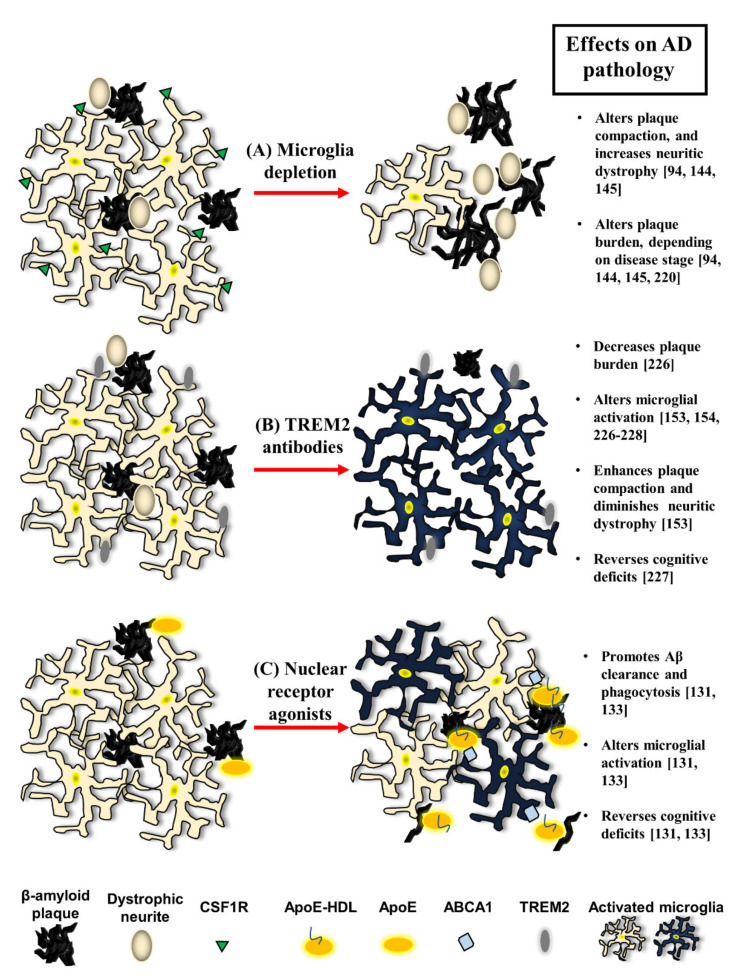
Microglia-directed manipulations and therapeutics modify pathology in AD. Microglia can be targeted through three generalized paradigms. (**A**) In microglia depletion models, CSF1R inhibitors kill microglia. Depletion can alter plaque compaction, enhance neuritic dystrophy and modify plaque burden depending on the disease stage. (**B**) TREM2 antibodies have been shown to promote microglial activation profiles amenable to phagocytosing plaques, enhancing plaque compaction, abrogating neuritic dystrophy and ameliorating cognition. (**C**) Nuclear receptor agonists promote upregulation of lipoprotein ApoE, its lipid transporter ABCA1 and production of ApoE-HDLs, the former of which promotes Aβ clearance by microglia. Nuclear receptors also enhance Aβ phagocytosis, alter microglia polarization and reverse cognitive deficits in various mouse models of AD. In this figure, references are denoted after the effects on AD pathology of each paradigm.

In amyloid-exclusive mouse models of AD, both disease progression and duration of CSF1R-inhibition dictate the impacts of microglia depletion on pathology (Figure 2A). Long-term microglial depletion (e.g., three or more months) prior to plaque deposition improves behavior and prevents plaque formation in the parenchyma, except in the vasculature where microglia persisted [144,220]. Acute depletion of microglia prior to plaque deposition is also neuroprotective, by rescuing dendritic spine and neuronal loss [213]. Microglial depletion at peak pathology in an AD mouse model reduces plaque burden, alters plaque morphologies and accelerates neuritic dystrophy, but these effects revert upon microglial repopulation [145]. In older AD mouse models, transient elimination of microglia does not affect Aβ burden, but neuroinflammation is reduced and certain behavioral tasks are improved [213]. The depletion of microglia does not impact behavior early in disease [144]. These varied outcomes illustrate the complexity of microglial function.

There is a possibility that repopulated microglia may be able to execute more efficient disease-modifying functions, but this could depend on the time course of the disease or the type of disease [145,213,221]. It should also be noted that CSF1R inhibition, or genetic models to deplete microglia, can alter peripheral myeloid and lymphoid cell population numbers in addition to microglia [222,223]. This finding could impact prior studies where significant input of the peripheral immune system occurred in the context of CNS disorders. Another point to consider is that microglia depletion models show enhanced cytokine storm and astrocytosis [13,94,216]. Some CSF1R inhibitors do not deplete microglia, but rather hinder microglial functions, such as proliferation, activation and migration—consistent with CSF1R inhibition in peripheral macrophages [63,224]. In some of these studies, the inhibition of microglia proliferation or activation did not alter plaque burden, but rather shifted plaque-associated microglia to an anti-inflammatory phenotype, corresponding to improved cognitive and behavioral outcomes in an AD mouse model [225]. Thus, microglia depletion in AD is a novel means to a therapeutic approach for AD, but also to uncover the mechanisms which underlie microglia-dependent processes in AD.

Targeting microglia specifically appears to be an attractive option. Specific microglial receptors, such as TREM2, provide an initial signal for DAM/MGnD activation by binding to ApoE [51,52,56]. Along these lines, recent studies have shown that humanized antibodies targeting TREM2 promote microglia activation, reduce pathology, correct cognitive deficits and dictate microglial responses even in AD mouse models carrying TREM2 variants [153,154,226,227,228]. This is particularly important, as some TREM2 variants, such as TREM2-R47H [126], are considered loss-of-function mutations and are associated with worsened pathology in AD mouse models [62]. The overexpression of TREM2 also ameliorates pathology and reverses cognitive deficits in an AD mouse model through microglia activation [181]. Therefore, therapeutics that recalibrate microglial activation through TREM2 are particularly attractive (Figure 2B).

### 6.3. Usage of Nuclear Receptor Agonists to Target Microglia

There is substantial literature demonstrating the salutary actions of therapeutics in AD mouse models. In particular, nuclear receptor agonists drive the transcription of genes associated with Aβ clearance and phagocytosis, and the modulation of inflammation in various mouse models of AD [131]. The nuclear receptor superfamily is composed of hundreds of receptors expressed ubiquitously in mammalian tissues [229]. One class of nuclear receptors form heterodimers exclusively with the retinoid X receptor (RXR). These RXR-heterodimers reside on DNA in the nucleus tethered to co-repressor proteins; once respective ligands bind, co-repressor proteins are exchanged with co-activator proteins, initiating target gene transcription [131]. As heterodimers with RXR, liver X receptors (LXRs) and peroxisome proliferator-activated receptors (PPARs) both activate genes associated with Aβ clearance, phagocytosis and the resolution of inflammation [133]. In various AD mouse models, nuclear receptor agonists broadly ameliorate AD pathology as well as rescue cognitive and behavioral deficits [131]. LXR activation promotes the upregulation of ApoE as well as its lipid transporters ABCA1 and ABCG1, leading to the formation of ApoE-HDL particles. In astrocytes and microglia, ApoE-HDLs promote the degradation and clearance of soluble Aβ [132]. Inflammatory signaling is also dampened, since nuclear receptors promote the transrepression of pro-inflammatory cytokines, such as IL-6 and TNFα, in AD mice and in microglia specifically [230,231,232,233,234]. PPAR signaling activates genes associated with lipid clearance, including *Lpl* and *CD36*. Phagocytic protein machinery molecules, such as Aβ-binding scavenger receptor CD36 [140], and phagocytic-like receptors such as MerTK or TREM2 [141,235], can also be induced via activation of nuclear receptor signaling (Figure 2C).

It should be noted that the salutary actions of nuclear receptor agonists in AD or homeostasis occur through other glia, and even directly in neurons as well [131,133,236,237]. Although nuclear receptor agonists act on all cell types of the brain, microglia preferentially express a broad repertoire of processes amenable to nuclear receptor agonist action [238]. Some markers of the DAM/MGnD phenotype that are responsible for lipid clearance are direct target genes of PPAR (e.g., *Lpl*) or LXR (e.g., *Apoe*), pointing to their collective importance in regulating microglial homeostasis during AD [239]. Interestingly, plaque-associated ApoE is largely sourced to microglia [185], but astrocytes are also proficient producers of ApoE during steady-state [240]. The overall impact of glial-specific (e.g., microglia or astrocytes) nuclear receptor signaling on AD pathology has yet to be demonstrated. This distinction may be critical going forward to develop therapeutics specific to microglia in both the AD and neurodegenerative disease arena.

## 7. Conclusions

In this review, we focused on the importance of microglia during homeostasis and neurodegenerative diseases, with particular focus on their functions during AD. The recent advances in ontological tracing and single-cell sequencing have rapidly expanded our understanding of microglial biology during development and disease. Microglia-specific receptors play key roles during AD progression, along with inflammatory mediators present on or released by microglia. In humans and in mouse models, DAM/MGnD phenotypes are broadly manifested across neurodegenerative landscapes, but it is unclear whether scientists and clinicians can exploit these cells to ameliorate disease with targeted therapeutics or other means. Although monocytes are rarely trafficked into the brain in AD mouse models, clearer evidence now exists concerning myeloid cells at the brain borders. As such, their similarities to microglia should not be ignored when considering the progression of neurodegenerative disorders. In concert, the pathological roles microglia play during AD are complex, and several questions remain unanswered. Nonetheless, microglia or their specialized functions as immune cells can be targeted with various therapeutics, leaving scientists with several means to uncover how these unique cells contribute to and regulate the homeostatic and disease processes of the CNS.

## Figures and Tables

**Table 1:** Overview of selected microglial receptors and signaling molecules during development, homeostasis and neurodegenerative diseases.

Receptor/*Ligand(s)*	Functions during Development and Homeostasis	Functions during Neurodegenerative Diseases
**CX3CR1**/*CX3CL1*	Synapse engulfment [18,46], and microglial migration. Blocks neuronal excitotoxicity [47]. KO show altered behavior [48], ↓ Layer V neurons [49].	KO animals show enhanced microglial-mediated plaque barriers, and ↓ neuritic dystrophy [50]. *Cx3cr1* downregulated in DAM/MGnD phenotypes [51,52]
**P2RY12**/*Adenosine and uridine nucleotides (e.g., ATP)*	Metabolite sensing, protrusion extension [17,53] and inhibits neuronal excitotoxicity with CD39 (*Entpd1*) [39]. Maintains microglial regulatory junctions between neurons [54]. Controls CA1 neuron excitability and fear memory [55].	*P2ry12* and *Entpd1* downregulated in DAM/MGnD phenotypes [51,52]. *Entpd1* KO show enhanced seizures [39].
**TREM2**/ *ApoE* [56]*, Aβ* [57]*, lipids* [56,58]	Enhances synapse elimination during development [43]. Aids in astrocytic engulfment of synapses during development [42]. KOs lack migratory and activation profiles [59].	Enhances microglial activation [60]. ↓ in microglial-plaque barrier and ↑ neuritic dystrophy in KOs [61,62]. ↑ homeostatic signature in KOs [52].
**CSF1R**/ *CSF-1, IL-34* [63]	Regulates neuronal differentiation and survival in neuronal progenitors [64]. KOs of CSF1R [65] or ligands [9,66] show ↓ microglia and other myeloid cell populations.	During experimentally-induced injury, CSF1R expression on neurons mediates their survival [67]. CSF1R mutations promote specific type of neurodegenerative disease [64]. Signaling modifies disease in AD [28,68].
**TGF-β1**/TGFβ-R1	Regulates C1q expression during development [37]. KO animals lack microglia [12] and ↓ synaptic plasticity [69]. KOs show ↓ motor behaviors [12].	CNS KO animals lack homeostatic signature [12]. In DAM/MGnD phenotypes, homeostatic signature is lost [52].
**C1q and C3**/ *C3R (binds C3 after conversion from C1q)*	C1q and C3 tag synapses for destruction during development [17,20]. ↑ synapses in KOs [17].	↑ C1q and C3 in AD, which leads to synapse loss and neurodegeneration [17,70]. Microglia-specific C1q drives neurotoxic astrocytes [71].
**CD200R**/*CD200*	CD200 expression on neurons and endothelial cells inhibits microglial activation in retina and brain [72,73]. CD200 KO mice show enhanced macrophage and microglia activation profiles [74].	CD200 upregulation during injury on neurons may protect against microglial-mediated neuronal damage [75,76]. In AD human brains, CD200/CD200R expression is downregulated [77], and IL-4 upregulates CD200R in microglia during neuroinflammation [78]. Overexpression of CD200 in the brain enhances neurogenesis and promotes Aβ clearance in an AD mouse model [79].
**TLRs**/ *various* [80]	Act as pattern-recognition receptors to detect pathogen-associated molecular patterns and stimuli associated with innate immune responses during homeostasis in the CNS [80,81].	TLR4 forms a complex with Aβ-binding co-receptor CD14, activating microglia [82,83]. Microglia lacking TLR4 show diminished phagocytosis of fAβ and release of inflammatory mediators [84]. Mutations in TLR4 enhance Aβ burden and reduce microglial activation in an AD mouse model [85].

Abbreviations: KO (knockout); ↑, ↓ (increased, decreased, respectively).

## Data Availability

Not applicable.
